# Addressing Missing Data in Patient‐Reported Outcome Measures (PROMS): Implications for the Use of PROMS for Comparing Provider Performance

**DOI:** 10.1002/hec.3173

**Published:** 2015-03-05

**Authors:** Manuel Gomes, Nils Gutacker, Chris Bojke, Andrew Street

**Affiliations:** ^1^ Department of Health Services Research and PolicyLondon School of Hygiene and Tropical MedicineLondonUK; ^2^ Centre for Health EconomicsUniversity of YorkYorkUK

**Keywords:** missing data, multiple imputation, patient‐reported outcome measures, provider performance, missing not at random

## Abstract

Patient‐reported outcome measures (PROMs) are now routinely collected in the English National Health Service and used to compare and reward hospital performance within a high‐powered pay‐for‐performance scheme. However, PROMs are prone to missing data. For example, hospitals often fail to administer the pre‐operative questionnaire at hospital admission, or patients may refuse to participate or fail to return their post‐operative questionnaire. A key concern with missing PROMs is that the individuals with complete information tend to be an unrepresentative sample of patients within each provider and inferences based on the complete cases will be misleading. This study proposes a strategy for addressing missing data in the English PROM survey using multiple imputation techniques and investigates its impact on assessing provider performance. We find that inferences about relative provider performance are sensitive to the assumptions made about the reasons for the missing data. © 2015 The Authors. *Health Economics* Published by John Wiley & Sons Ltd.

## 1 Introduction

Non‐response is a major concern in health surveys because individual non‐respondents tend to be systematically different from those providing complete data (Cohen and Duffy, 2002; Perneger *et al*., 2005; Schenker *et al*., 2006). The reasons for the non‐response are rarely completely independent from both observed and unobserved values, meaning that data are not missing completely at random (MCAR). Consequently, inferences based solely on the respondents will be misleading. If the differences between respondents and non‐respondents can be explained entirely by differences in the observed data, such as characteristics of the patients, data are said to be missing at random (MAR). If so, it is possible to condition analyses on observed factors, thereby correcting the bias caused by missing data. However, differences between respondents and non‐respondents may depend on unobserved values, in which case data are missing not at random (MNAR). If missingness is associated with unmeasured factors, conditioning on the observed data will not eliminate entirely potential bias.

One area where missing data have recently raised important concerns is in the assessment of hospital performance (Gale *et al*., 2011; Groene *et al*., 2014; Kirkham, 2008). Missing data may bias performance assessments through several routes. Firstly, within each provider, individuals with complete information tend to be systematically different from those with missing data. Secondly, provider assessments that are based on smaller samples will lead to increased uncertainty in the estimation of provider effects. Indeed, providers with large proportions of non‐response will be less likely to be identified as statistically significantly better or worse than the benchmark. Thirdly, the reasons for the missing data may be related to the provider, not just the patient. For example, hospitals may differ in their data collection according to observed characteristics such as their volume of activity and their staffing arrangements (Hutchings *et al*., 2014; McCall *et al*., 2004).

The English patient reported outcome measure (PROM) programme involves collecting survey responses from patients in order to facilitate comparative performance assessment of different healthcare providers. Since April 2009, all providers of publicly funded inpatient care in the National Health Service (NHS) have been required to collect both generic and condition‐specific PROMs for four elective procedures: unilateral hip and knee replacements, varicose vein surgery, and groin hernia repairs. Patients having these procedures are invited to report their health status before and three or six months after surgery using paper‐based questionnaires. By comparing these before and after measures, changes in health can be identified and used to better understand differences in the systematic effect that health providers have on their patients' health (Appleby and Devlin, 2004). However, as with other health surveys, patients are not obliged to participate so responses will be missing if some fail to do so. Data may also be missing because providers differ in the effort they exert in overseeing data collection. Indeed, their efforts in this regard may be linked to their performance in terms of improving their patients' health status. Originally, the English PROM programme did not include a direct mechanism to police data collection, thereby opening up the possibility of gaming, either to lower the cost of data collection or to avoid reputational damage.

Recent incentive schemes linking payments to achievements in terms of health improvements have explicitly contemplated the issue of missing data. The English ‘best practice tariff’ (BPT) pay‐for‐performance scheme, based on PROM data and which started in April 2014, comprises two components (Monitor, 2013). First, providers qualify for bonus payments if they do not perform statistically significantly below a national benchmark with respect to risk‐adjusted improvements in patients' health status. Second, in order to receive this bonus, providers must ensure that they collect PROMs for over 50% of the patients eligible for the survey. This policy creates an incentive for providers to meet the minimum standard for data collection, but this does not necessary eliminate the problem of missing data (Gutacker *et al*., 2015). An important concern is whether the assessment of hospital's relative performance crucially hinges on the assumptions made about the reasons for the missing data.

This paper presents a strategy for addressing the missing data in PROMs and assesses its impact on the use of PROMs for comparing provider performance. Here, we consider multiple imputation (MI) methods that offer particular advantages for addressing missing data in performance assessments compared with other commonly used approaches such as maximum likelihood and inverse probability weighting. In the next section, we describe the data and the different missing data patterns in the PROM survey. Section 3 presents the methods for estimating provider‐specific outcomes and illustrates the implications of non‐response for reporting provider performance. Section 4 describes the approach for dealing with the missing data under MAR and sensitivity analyses to investigate potential departures from MAR. Section 5 reports the results on provider performance according to different assumptions made about the missing data. The last section discusses the findings and highlights some priorities for future research.

## 2 PROM Data

Our sample includes all patients aged 12 or over, who underwent primary, unilateral hip replacement surgery during the period of April 2011 to March 2012. All providers of publicly funded inpatient care in the English NHS are required to offer a pre‐operative PROM questionnaire (Q1) to all patients deemed fit for surgery. Patients complete this questionnaire, usually during the last outpatient appointment preceding the surgery or on the day of admission. Patients are surveyed again approximately six months after surgery via another questionnaire sent by mail (Q2). This post‐operative questionnaire is administered at the national level by an organisation contracted by the Department of Health.

Patient's health status before and after surgery is measured using a condition‐specific measure, the Oxford Hip Score (OHS), and a generic quality‐of‐life measure, the EuroQol‐5 dimensions (EQ‐5D). Here, we focus on the former given that it is used for BPT arrangements (Monitor, 2013). OHS consists of 12 components (questions) on different aspects of hip problems such as pain and functioning (Dawson *et al*., 1996; Murray *et al*., 2007). Each component has five possible responses, scored from 0 (most severe symptoms) to 4 (no symptoms). The overall score is a simple unweighted sum of all individual components, ranging from 0 (most severe level and highest number of symptoms) to 48 (least symptoms).

Administrative data about all patients having hip replacement, irrespective of whether they completed a PROM survey, are available in the Hospital Episodes Statistics (HES). HES includes detailed patient‐level hospital records about all NHS‐funded inpatient care provided by public and private hospitals in England. HES data are linked to the PROM survey responses through a matching variable provided by the Health and Social Care Information Centre (HSCIC). This linkage allows us to (i) ascertain the full population of hip replacement patients who were eligible to complete the PROM survey and (ii) obtain important clinical and socio‐demographic information about the patients that would not otherwise be available in PROMs.

The PROM data collection and linkage process may result in different types of missing data. For example, Q1 may be missing because (i) the HSCIC was unable to match the Q1 PROM record to the HES episode, because the requisite information for matching was missing (e.g. NHS number); (ii) the provider failed to administer the Q1 questionnaire; or (iii) the patient refused to complete it. The post‐operative Q2 questionnaire was sent only to those patients who answered the Q1 questionnaire, even if some answers were incomplete (item non‐response). Even though patients provided a Q1 questionnaire, the Q2 questionnaire might also feature missing data, either because the patient failed to return it or because some questions were left unanswered.

## 3 Missing Data in the Assessment of Provider Performance

### 3.1 Estimation of provider‐specific outcomes

For provider‐specific outcomes to be comparable, adjustment for the different case mix of patients within each provider is required. This is typically undertaken using a regression framework, which we will denote as the *analysis* model. In this paper, we built upon the NHS case‐mix adjustment methodology to estimate provider‐specific outcomes (Nuttall and Parkin, 2013). Let *y*_2,*ij*_ be the post‐operative observed health outcome for patient *i* treated in provider *j*. We adjusted *y*_2,*ij*_ for key patient characteristics (*X*_*ij*_), such as age, gender, co‐morbidities and socio‐economic status (measured using an index of multiple deprivation), as well as the pre‐operative health outcome (*y*_1,*ij*_). The analysis model is defined as

(1) $$\begin{array}{cc} \begin{array}{cc} y_{2,ij}=\alpha +X_{ij}\beta +y_{1,ij}\gamma +u_{j}+{\varepsilon_{i}}_{j,} & \varepsilon_{ij}∼ \end{array}N\left(0,\sigma_{\varepsilon }\right) & u_{j}∼N\left(0,\sigma_{u}\right) \end{array}$$

Both the provider‐specific unobserved effects (*u*_*j*_) and the error term (*ε*_*ij*_) are assumed to be normally distributed with zero mean and constant variance. Provider‐specific effects can be estimated using a fixed effect or random effect model. Here, we considered the latter as it is typically more efficient.^1^ To estimate how provider *j* performs relative to the national average, we considered an indirect standardisation approach recommended by the NHS case‐mix adjustment (Nuttall and Parkin, 2013):

(2) $$\begin{array}{cc} {\tilde{y}}_{2,j}=\rho_{j}{\bar{y}}_{2,} & {\hat{\rho }}_{j}=\frac{1}{n}\sum_{i=1}^{n}\left(\frac{y_{2,ij}}{{\hat{y}}_{2,ij}}\right) \end{array}$$

The adjusted provider‐specific outcome $\left({\tilde{y}}_{2,j}\right)$ is obtained by multiplying the national average outcome $\left({\bar{y}}_{2\text{,}}\right)$ by a provider‐specific factor $\left({\hat{\rho }}_{j}\right)$ that reflects the extent to which the provider's observed outcome (*y*_2,*ij*_) compares with its expected outcome (*ŷ*_2,*ij*_).

### 3.2 Funnel plots and missing data

**Figure 1:**
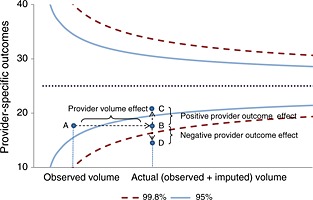
Missing data and its implications for assessing provider performance via funnel plots

It is common to compare provider performance using funnel plots (Department of Health, 2012b; Spiegelhalter, 2005). Here, outcomes are plotted against volume (the number of patients treated) with 95 (2 standard deviations) and 99.8% (3 standard deviations) control limits used to indicate those providers that perform better or worse than expected (Figure 1). Accordingly, those providers located above the 95 and 99.8% control limits are judged to have a positive *alert* and *alarm* status, respectively, while negative *alerts* and *alarms* are those located below the 95 and 99.8% control limits, which are often under greater scrutiny. The pay‐for‐performance BPT scheme requires that providers do not perform statistically significantly below the national average, i.e. being located outside the 99.8% control limit (negative *alarm*), to be eligible for bonus payments. The BPT guidance sets out the requirement to use funnel plots to report provider performance, although other approaches such as caterpillar plots or z‐scores are also available (Goldstein and Spiegelhalter, 1996).

With missing outcomes, the assessment of provider performance requires careful consideration. Ideally, we would like to remove the impact of non‐response from inferences about provider performance, in order to locate the hospital near its ‘true’ mean outcome and volume on the funnel plot. Consider a hospital with a low response rate such that its observed mean outcome and volume are at point A (*in control*) on a standard funnel plot (Figure 1). Instead, if all patients were observed for this hospital, and the unobserved outcomes were similar to those observed (data were MCAR), then the hospital would be located at point B (negative *alert*). In this particular stylised example,^2^ because of the missing data, the provider would be judged to be *in control* rather than a negative *alert*. From the hospital's point of view, this is a more desirable status, but such an assessment is misleading.

The assumption that the patients with observed outcomes are similar to those who have missing outcomes (MCAR) is unlikely to hold. Missing data will be dependent on observed factors (MAR), other than those included in the analysis model, and might depend on unobserved values (MNAR). Therefore, the handling of missing data under MAR or MNAR may have four main effects for assessing provider performance via funnel plots.^3^ The first two effects refer to the location of the provider‐specific effect and the other two to the placement of the population benchmark and control limits:

- **Provider volume effect**. This will always shift the provider location to the right, for example, from point A to B (Figure 1). *Ceteris paribus*, moving from the observed to the actual volume increases the probability of being located outside the control limits.
- **Provider mean outcome effect**. Under MAR or MNAR, the provider‐specific mean outcome may shift downwards or upwards depending on whether the outcomes for all provider's patients (observed and imputed) are better or worse than the observed outcomes. In Figure 1, the hospital would move from point B to C (*in control*) if the patients for whom data are missing have a relatively better profile than those for which data are observed, or to point D (negative *alarm*) otherwise (Figure 1).
- **Population variability effect**. The control limits will be narrower or wider depending on whether the variability of outcomes in the full population is smaller or larger than that for the sample of patients whose outcomes are observed.
- **Population mean outcome effect**. The overall mean outcome (horizontal line) will move up or down, according to whether the actual outcomes for the entire population are, on average, better or worse than the observed outcomes.

The impact of addressing the missing data on the location of the provider in the funnel plot will be mainly determined by (i) the provider's volume effect and (ii) how different its missing outcomes are from those observed (the provider mean outcome effect), although (iii) and (iv) will also have some effect on that.

## 4 Methods for Addressing the Missing Data

### 4.1 Complete case analysis

A common approach for dealing with missing data is to discard patients for whom any outcome or covariate is missing. While complete case analysis (CCA) is simple to implement, this approach is only valid when data are MCAR. This implicitly assumes that individuals with complete data are representative of those with missing data, conditional on the variables included in the analysis model. The current official approach to performance assessment using PROMs is to apply CCA.

### 4.2 Multiple imputation

With MI, each missing value is replaced by a set of plausible values, which are drawn from the posterior distribution of the missing outcomes given the observed data. Standard implementation of MI assumes that the probability of observing the outcomes is independent of any unobserved values, given the observed data (MAR). After imputation, the analysis model is applied to each multiple imputed dataset to estimate the parameters of interest. The multiple imputed estimates are typically combined using Rubin's rules (Rubin, 1987), which properly reflect the variation between and within imputations.

A key feature of MI is that the model for the missing data is estimated separately from the analysis model for estimating the parameters of interest. This allows us to include in the imputation model *auxiliary* variables in addition to those used in the analysis model that are associated with both the outcome and missingness. This is an important advantage of MI when compared with commonly used maximum likelihood approaches, which makes a potentially stronger MAR assumption that all observed factors that give rise to the missing data are included in the pre‐specified analysis model. Including these *auxiliary* variables in the imputation model can reduce bias, improve precision and help make the MAR assumption more plausible. An additional advantage of MI is that its framework naturally extends to the assessment of alternative assumptions about the missing data mechanism (Section 3.2).

For MI to provide valid inferences, the imputation model must accommodate the structure and the distribution of the data. In PROMs, the imputation model needs to recognise that the probability of non‐response may be more similar within than across providers. Indeed, missingness may depend on observed patient‐level characteristics that tend to be more similar within the provider, and on provider‐level characteristics such as whether the provider is a public (NHS) or private hospital. Compatible with the analysis model, this can be achieved by including provider‐specific random effects (Gomes *et al*., 2013).

Typically, MI assumes normality for continuous outcomes, but the post‐operative OHS is left skewed with a spike at 48. Finding a suitable transformation to help normalise this outcome can be difficult. An alternative approach is to address the missing data in the individual components of the OHS that are ordered (0 to 4). With ordinal components, we can consider a latent normal variable for each component of the score. An important advantage of the latent normal approach (Albert and Chib, 1993), which is equivalent to the probit model, is that it naturally links with the multivariate normal imputation model, easily implemented in standard software (Carpenter *et al*., 2011). Hence, we can impute these latent variables, assuming that their variance is restricted to one, along with other continuous variables, for example missing covariates.

Let $h_{ij}^{k}$ be the observed *k*th component (*k* = 1, …, *K*), with *M* ordinal categories (*m* = 1, …, *M*), of the self‐reported OHS score for individual *i* in provider *j* (for the OHS score, *K* = 12 and *M* = 5). Let $\pi_{ij,m}^{k}=\mathrm{Pr}\left(h_{ij}^{k}=m\right)\text{and}\;\gamma_{ij,m}^{k}=\mathrm{Pr}\left(h_{ij}^{k}\leq m\right)$. By considering the ordered probit link model (Green, 2003), probit $\left(\gamma_{ij,m}^{k}\right)=\Phi^{-1}\left(\gamma_{ij,m}^{k}\right)=\alpha_{m}^{k}$ , then $h_{ij}^{k}$ can be described as a latent normal variable, $Z_{ij}^{k}\sim N\left(0\text{,}1\right)$, with the following threshold model:

(3) $$h_{ij}^{k}=\left\{\begin{array}{c} 0\;\text{if}\;Z_{ij}^{k}\leq \;\alpha_{1}^{k}\\ m-1\;\text{if}\;\alpha_{m-1}^{k}<Z_{ij}^{k}\leq \alpha_{m}^{k},\;m=2,\ldots ,M-1\\ 4\;\text{if}\;Z_{ij}^{k}>\alpha_{M-1}^{k} \end{array}\right.$$

The threshold parameters, $\alpha_{m}^{k}$, define the *m*th category of the component *k*. The multivariate latent model is then given by ***Z***_*ij*_ = ***βX***_*ij*_ + ***u***_*j*_ + ***e***_*ij*_, with

$$\begin{array}{cc} e_{ij}\sim N\left[0,\Omega_{e}=\left(\begin{array}{ccc} 1 & \cdots & \rho \\ \vdots & \ddots & \vdots \\ \rho & \cdots & 1 \end{array}\right)\right] & u_{j}\sim N\left[0,\Omega_{u}=\left(\begin{array}{ccc} \tau_{1}^{2} & \cdots & \phi \tau_{1}\tau_{K}\\ \vdots & \ddots & \vdots \\ \phi \tau_{K}\tau_{1} & \cdots & \tau_{K}^{2} \end{array}\right)\right] \end{array}$$

where $Z_{ij}=Z_{ij}^{1},\;\ldots ,Z_{ij}^{K}$, $X_{ij}=X_{ij}^{1},\;\ldots ,X_{ij}^{K}$ are the auxiliary variables, $u_{j}=u_{j}^{1},\;\ldots ,u_{j}^{K}$ are the provider‐specific random effects and $e_{ij}=e_{ij}^{1},\;\ldots ,e_{ij}^{K}$ are the error terms. The level 1 variance ( $\sigma_{k}^{2}$) is constrained to 1. The level 2 correlation (*ϕ*) is often set to zero to avoid over‐parameterisation at level 2. The Markov chain Monte Carlo (MCMC) algorithm to impute *k* ordinal components is provided in Appendix 1.

We implemented a distinct imputation model for missing data pattern at Q1 and Q2. Missing covariates such as ethnicity and duration of symptoms were jointly imputed with the incomplete outcome. Both imputation models included all predictors included in the analysis model, and a number of auxiliary variables identified in previous studies (Gutacker *et al*., 2015; Hutchings *et al*., 2012; Hutchings *et al*., 2014) that were strong predictors of missingness and associated with the post‐operative outcome. More specifically, in the imputation model for missingness pattern at Q1, we have included two patient‐level auxiliary variables: (i) hospital length of stay and (ii) elective waiting time, and three provider‐level characteristics: (i) whether the provider was an NHS or private provider; (ii) whether the provider was a teaching hospital; and (iii) surgery volume of the hospital. In the imputation model for missingness pattern at Q2, we have included the following patient‐level auxiliary variables: (i) hospital length of stay; (ii) elective waiting time; (iii) a dummy variable to indicate previous hip replacement; (iv) a dummy to indicate Q1 was administered before hospital admission; (v) whether assistance was required in completing Q2; and (vi) whether the patient lived alone. No provider‐level variables were included in this imputation model.

We conducted 100 imputations and 10 000 MCMC iterations, with each set of imputed values being drawn from the posterior distribution at every 100th iteration of the MCMC chain. After imputation, we combined the individual OHS components into an overall OHS score. Then, we applied the analysis model (model (1)) to each multiple imputed dataset to estimate our parameter of interest, the adjusted provider‐specific post‐operative OHS (model (2)) and combined the results using Rubin's rules. All analyses were implemented in Stata, version 13, with imputations conducted in the software REALCOM‐impute called from Stata (Carpenter *et al*., 2011).

### 4.3 Sensitivity analysis

The approach taken to handling missing data requires careful consideration of the different reasons as to why they are missing (Little and Rubin, 2002). Because the true missing data mechanism is unknown, it is important to examine whether inferences about comparative provider performance are robust to alternative assumptions concerning the reasons for the missing data. A practical approach is to undertake sensitivity analyses after MI under MAR. Here, we considered a weighting approach after MI that uses the concept of importance resampling (Carpenter *et al*., 2007).

Let *R*_*ij*_ = 1 if the outcome (*y*_2,*ij*_) is observed, 0 otherwise. Let the probability of observing the outcome, *P*(*R*_*ij*_ = 1), depend on observed patient (*W*_*ij*_) and provider (*Z*_*j*_) characteristics but also on the underlying outcome, *y*_2,*ij*_.

(4) $$P\left(R_{ij}=1|W_{ij},Z_{j},y_{2,\;ij}\right)=\eta_{0}+W_{ij}\eta_{1}+Z_{j}\eta_{2}+\phi_{j}+y_{2,ij}\delta +\epsilon_{ij}$$

Equation (4) collapses to a MAR mechanism when *δ* = 0. The basic idea is to explore the sensitivity of the results as *δ* departs from 0. After we have generated *M* multiple imputed datasets under MAR, we apply the analysis model to each dataset and obtain *M* estimates. Then, instead of a simple average, a weighted average is computed after assigning a relatively higher weight to those imputations judged to have a more plausible MNAR mechanism (for a chosen *δ*).

Suppose we order the data so that patients *i* = 1, …, *n*_1_ have missing outcomes and patients *i* = *n*_1 + 1_, …, *N* have complete data. For patients with incomplete data, let $y_{ij}^{m}$ denote the *m*th imputed value under MAR, *m* = 1, 2, …, *M*. Under the logistic model for the missingness model described in model (4), Carpenter and others (Carpenter *et al*., 2007) showed that the weights can be a simple function of the imputed data and the chosen *δ*. Given that *δ* represents the log‐odds ratio of the chance of observing *y* for each unit change in *y*, then the weight for imputation *m* can be calculated as ${\tilde{w}}_{m}=\exp\left(-\delta \sum_{i=1}^{n1}y_{i}^{m}\right)$ 4. For each imputed dataset, we can obtain the relative weight by normalising them as follows: $w_{m}={\tilde{w}}_{m}/\sum_{i=1}^{m}{\tilde{w}}_{m}$.

Then, under the MNAR model implied by *δ*, the *M* parameters of interest ( ${\tilde{y}}_{2,j}$) are combined using Rubin's rules, but with each imputation being re‐weighted according to the relative weight (*w*_*m*_) as follows (under MAR, all imputations are equally weighted):

(5) $${\tilde{y}}_{2,j}^{\text{MNAR}}=\sum_{m=1}^{M}w_{m}{\tilde{y}}_{2,j}\;Var_{{\tilde{y}}_{2,j}}^{\text{MNAR}}={\tilde{V}}_{W}+\left(1+\frac{1}{M}\right){\tilde{V}}_{B}$$

where ${\tilde{V}}_{W}=\sum_{m=1}^{M}w_{m}{\hat{\sigma }}_{m}^{2}$ is the within‐imputation variance, and ${\tilde{V}}_{B}=\sum_{m=1}^{M}w_{m}{\left({\tilde{y}}_{2,j}-{\tilde{y}}_{2,j}^{\text{MNAR}}\right)}^{2}$ is the between‐imputation variance.

The weights provide, therefore, a simple mechanism to correct (re‐weight) those imputations judged to have a less plausible missing data mechanism across alternative departures from MAR. For example, when *δ* is positive, the probability of observing *y* is higher for patients reporting better health (more positive *y*). This means that for imputations under MAR, patients reporting poorer outcomes will be under‐represented. The weights correct for this by up‐weighting the estimates from those imputations where the sum of the imputed values of *y* is small.

## 5 Results

Table 1 reports descriptive statistics for the outcome, the risk adjustment predictors included in the analysis model and the auxiliary variables used in the imputation model, for our sample of individuals undergoing elective hip replacement surgery in 2011–2012. Most patients were women, over 55 years old and white. Typically, these patients had 0 or 1 co‐morbidities and symptoms for 5 years or longer. On average, patients had a substantial health improvement six months after surgery with mean OHS more than doubled. The overall proportion of individuals with incomplete outcomes and covariates was 48%.

**Table 1:** Descriptive statistics of outcome, risk adjustment predictors and auxiliary variables used in the imputation models, for individuals undergoing hip replacement in 2011–2012 (N = 71 821)

Variable	N (%) or Mean (SD)	% observed
Outcome		
Post‐operative OHS	38.1 (9.5)	52%
Risk‐adjustment predictors		
Pre‐operative OHS	17.5 (8.4)	61%
Male	28 979 (40%)	100%
Age		100%
*Under 55*	8694 (12%)	
*55–65*	15 736 (22%)	
*65–75*	25 133 (35%)	
*Over 75*	22 376 (31%)	
Ethnicity (Non‐White)	8027 (13%)	89%
Charlson Comorbidity Index		
*0*	51 957 (72%)	
*1*	15 213 (21%)	
*2 or more*	4769 (7%)	
Index of Multiple Deprivation quintile		100%
Most deprived	9163 (13%)	
*2nd*	12 468 (17%)	
*3rd*	16 524 (23%)	
*4th*	18 399 (26%)	
*Least deprived*	15 166 (21%)	
Duration of symptoms		61%
*Up to 1 year*	6182 (14%)	
*1–5 years*	29 365 (67%)	
*Over 5 years*	8479 (19%)	
Patient‐level auxiliary variables		
Hospital length‐of‐stay (days)	6.01 (7.51)	100%
Waiting time (days)	88 (61)	100%
Previous surgery	39 690 (89%)	38%^a^
Q1 administered before admission	36 543 (82%)	38%^a^
Assisted in completing Q2	41 606 (94%)	38%^a^
Living alone	11 615 (26%)	38%^a^
Provider‐level auxiliary variables (N = 298)		
Private	147 (49%)	100%
Teaching hospital	32 (11%)	100%
Surgery volume	241 (250)	100%

a These auxiliary variables were taken from the PROM dataset, and they were missing for all patients for whom Q1 was missing. We have included these in the imputation model for missing pattern at Q2. For the subset of patients, these variables were fully observed, and therefore, we did not need to impute them.

**Figure 2:**
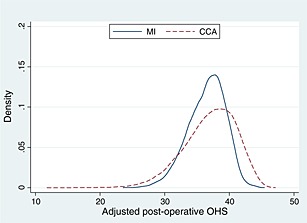
Kernel density of the risk‐adjusted post‐operative OHS for CCA and MI

Figure 2 shows the distribution of the post‐operative OHS after risk adjustment, for both the complete cases and after imputation. MI led to a slightly lower mean and smaller standard deviation) post‐operative OHS when compared with CCA: 37.3 (3.26) versus 38.1 (3.95). This suggests that patients with missing outcomes were associated with a somewhat poorer profile according to observed factors included in the imputation model.

**Figure 3:**
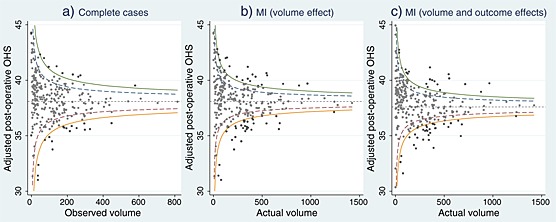
Funnel plots of provider‐specific outcomes according to complete cases (N = 279), and after multiple imputation: volume and mean outcome effects (N = 298)

Figure 3 illustrates the funnel plots of the provider‐specific OHS according to CCA and MI. The width of the control limits is similar between the two approaches. That is, conditional on the observed data, the variability in the individual outcomes is relatively similar between the observed sample and full population of patients. The proportion of providers performing statistically above and below the national average (according to both 95 and 99.8% control limits) is higher after MI. The changes in the performance status appear to be dominated by the volume effect, as illustrated in Figures 3b and 3c. The volume effect (Figure 3b) was obtained by plotting observed provider mean outcomes (i.e. assuming MCAR) against the provider's total volume (observed and imputed).

Table 2 reports the number of providers under each performance category according to CCA and MI. Overall, CCA leads to type‐II errors by failing to detect statistically significant overperformers (positive *alarms*) and underperformers (negative *alarms*). For example, the proportion of negative *alarms* (outside the lower 99.8% control limit) varied from 7 (N = 20) with complete cases to 11% (N = 32) after MI. Most of the providers that moved to an *alarm* status after MI were previously judged *alerts* based on CCA (more details are available in Appendix A1 in the supporting information). Under the new BPT, the number of providers who would be ineligible for a bonus (based on their performance status and the minimum 50% response rate) scheme was 67 (22%) according to CCA and 78 (26%) after MI (Appendix A1).

**Table 2:** Provider performance status according to CCA and MI

	CCA		MI (volume effect)		MI (volume and outcome effects)	
	**N**	%	**N**	%	**N**	%
Negative *alarm*	20	7.2	32	10.7	32	10.7
Negative *alert*	22	7.9	27	9.2	24	8.1
*In control*	214	76.7	185	61.9	187	62.8
Positive *alert*	14	5.0	32	10.7	35	11.7
Positive *alarm*	9	3.2	22	7.4	20	6.7
Total	279	100.0	298	100.0	298	100.0

PROMs were entirely missing for 19 providers, and hence, these were not assessed under CCA.

Table 3 reports the results of the sensitivity analysis. The relative provider performance fairly remained similar for values of *δ* below −0.3 and above 0.3, as a relatively lower number of imputations receive high weights. For example, *δ* = 0.3 means that the odds of response is 1.35 (exp(0.3)) times greater for patients with an additional unit in the post‐operative OHS. There is some evidence that patients reporting poorer outcomes were somewhat under‐represented in the imputations under MAR. Indeed, for positive values of *δ*, there was a relatively higher number of providers with negative *alarm* status and fewer providers with a positive *alarm* status. The distribution of the relative provider performance remained fairly unchanged for negative values of *δ*, i.e. when there is a negative association between the probability of observing the outcome and the outcome level.

**Table 3:** Sensitivity analyses to departures from MAR represented by alternative MNAR mechanisms

	Performance status according to OHS				
	Positive *alarm*	Positive *alert*	*In control*	Negative *alert*	Negative *alarm*
MNAR, *δ* = − 0.3	22 (7.4%)	28 (9.5%)	193 (65.4%)	22 (7.5%)	31 (10.5%)
MNAR, *δ* = − 0.2	21 (7.1%)	29 (9.8%)	192 (65.1%)	23 (7.8%)	31 (10.5%)
MNAR, *δ* = − 0.1	18 (6.1%)	28 (9.5%)	193 (65.4%)	22 (7.5%)	34 (11.5%)
MAR, *δ* = 0	20 (6.7%)	35 (11.7%)	187 (62.8%)	24 (8.1%)	32 (10.7%)
MNAR, *δ* = 0.1	14 (4.8%)	23 (7.8%)	192 (65.1%)	26 (8.8%)	40 (13.6%)
MNAR, *δ* = 0.2	13 (4.4%)	21 (7.1%)	191 (64.8%)	21 (7.1%)	43 (14.6%)
MNAR, *δ* = 0.3	13 (4.4%)	18 (6.1%)	195 (66.1%)	25 (8.5%)	44 (14.9%)

## 6 Discussion

The English PROM survey provides valuable evidence about the effectiveness of the health care delivered by the NHS in terms of health improvements to patients. An important aim of this programme is to support evaluations of the relative performance of providers in undertaking elective procedures such as hip replacement. As with other self‐reported, voluntary health surveys, PROMs are prone to large proportions of missing data because of reasons that are associated with both the patient and the provider. However, existing official assessments of the relative provider performance that use PROMs are based solely on the complete cases.

This study is a first attempt to address the implications of missing data for the use of PROMs in the assessment of provider performance. We present a strategy for addressing the missing data using MI methods and illustrate the impact of using such methods in reporting comparative performance. A major finding of the paper is that judgments about provider performance differ according to the assumptions made about the reasons for non‐response. We found that analyses according to complete cases led to a substantially lower number of providers performing statistically above or below the national average. By ignoring the problem, CCA assumes that the missing data are unrelated to both observed and unobserved values, conditional on the risk‐adjustment predictors. Previous studies have shown that this assumption is implausible in the context of PROMs (Gutacker *et al*., 2015; Hutchings *et al*., 2012; Hutchings *et al*., 2014).

We propose a strategy for handling the missing data using MI methods. MI is particularly suitable for addressing non‐response in PROMs for several reasons. Firstly, the model for the missing data is estimated separately from the analysis model. This offers particular advantages when compared, for example, with standard maximum likelihood approaches, because it allows the imputation model to include auxiliary variables that are predictive of missingness, without having to modify the pre‐specified risk adjustment model. Secondly, MI provides a flexible framework for assessing the sensitivity of the results to departures from MAR. Here, we considered sensitivity analysis by re‐weighting, but alternative approaches such as pattern mixture models are also available (Carpenter and Kenward, 2013). Thirdly, missing data patterns observed in PROMs are non‐monotone. Under this pattern, MI methods are preferable to alternative practical approaches such as inverse probability weighting (Carpenter *et al*., 2006). Fourthly, the MI approach fits well with the method used for reporting comparative provider performance. It facilitates identification of provider's performance status in the funnel, given its estimated mean outcome (under MAR) and actual (observed and imputed) volume.

Our findings have important implications for policy making. Firstly, given that the proportion of observed PROMs varies considerably across providers, appropriate mechanisms for improving data collection are warranted. If the provider expects to be an underperformer, it may have little incentive to have a higher response rate because that makes them more likely to be identified as such. This is because the uncertainty around the provider effect, as reflected in their confidence intervals or, equivalently, the control limits, is an inverse function of the sample size. We have illustrated this using funnel plots, but reporting provider performance via alternative methods such as caterpillar plots would reach a similar conclusion; lower response rates makes below‐average provider effects more imprecise and hence not statistically different from zero. The strong correlation of provider response rates over time (Gutacker *et al*., 2015) may be suggestive of gaming, but that could simply be as a result of a poor approach to data collection in some providers, while others are much better organised. By imposing a 50% (or above) response rate criterion in order to qualify for a bonus payment for good performance, the BPT initiative provides stronger incentives for providers to improve their process of data collection. However, this may not eliminate the problem entirely as providers may be induced to collect just enough data to satisfy the requirement, still leading to a potentially large proportion of non‐response and potentially unrepresentative samples. Increasing the threshold above 50% in the future can encourage providers to continually improve data collection.

Secondly, providers with small volumes do not show a different missing data pattern from that of the providers with large volumes. More importantly (and perhaps surprisingly), the relative performance of the providers does not seem to be associated with their volume of surgery.

Thirdly, the sensitivity analysis suggested that conditional on the observed data, individuals with poorer outcomes may be somewhat under‐represented. However, it is not possible to determine from the data whether this is because of unobserved factors related to the provider (e.g. gaming) or the patient.

Fourthly, commentators suggest that provider‐level characteristics should not be included in the model for estimating provider‐specific outcomes if they do not constitute binding production constraints (Smith and Street, 2005). However, with missing data, these variables should be carefully considered in the specification of the imputation model in order to minimise any potential bias because of the differences between the providers with different proportions of observed PROMs.

This paper has a number of limitations. First, we restricted our sample to the last available data cohort for hip replacement patients. Previous studies showed that non‐response was higher in previous cohorts and other elective interventions (Gutacker *et al*., 2015; Hutchings *et al*., 2014), and so dealing with missing data would be likely to have greater implications for the inferences on provider performance for these patient groups.

Second, our approach was to assign all patients to the provider of care reported in the HES inpatient record. However, care is sometimes subcontracted to private providers (Independent Sector Treatment Centres) and the assignment of patients to providers may therefore be incorrect. Given that NHS providers can subcontract a proportion of their activity with multiple independent centres, it is impossible to be precise about where treatment actually took place. We therefore acknowledge that our assessment of provider response rates relies on the assumption that provider codes have been recorded correctly. However, the impact on the estimated response rates is likely to be small (Hutchings *et al*., 2014).

Third, we have focused our an analysis on the disease‐specific OHS as this is the preferred outcome measure in the pay‐for‐performance BPT scheme. The impact of addressing missing data for the assessment of provider performance was very similar for the generic quality‐of‐life measure, the EQ‐5D. Results for this outcome can be found in Appendix A2 in the supporting information.

Fourth, throughout, we assumed that the imputation model was correctly specified. We followed methodological guidance and specified an imputation model that was compatible with the analysis model, for example, by including random effects and non‐linear interactions (Carpenter and Kenward, 2013; White *et al*., 2011). However, the use of methods that are less sensitive to the correct specification of the imputation model, such as Robust MI, may warrant consideration (Daniel and Kenward, 2012).

In conclusion, our analysis shows that inferences on the relative provider performance using PROMs differ according to the assumptions made about the missing data. Assessments based solely on observed PROMs are shown to underestimate the proportion of poorly performing providers. We provide a strategy for addressing the missing data that makes more plausible assumptions about non‐response given the observed data. Considerable attention has been given to investigating the factors associated with patient non‐response in health surveys. However, the reasons why health providers may differ in their ability and willingness to collect data are less well understood and should be investigated further in the context of PROMs and similar initiatives such as the English Friends and Family test (Department of Health, 2012a). In addition, future efforts are best invested towards increasing provider response rates so that inferences on provider performance are less dependent on modelling assumptions.

## Conflict of Interest

The authors have declared that there is no conflict of interest.

## Supporting information

Supporting info item

## Footnotes

1 Nuttall and Parkin (2013) reports coefficient estimates obtained using both fixed and random effect estimators. While the Hausman test rejects the null hypothesis that the random effect estimator is unbiased, the results on comparative provider performance are similar between the two approaches.

2 For instance, we have assumed that the missing values from this provider have no impact on the mean and variance of the overall outcome distribution.

3 All these effects also apply for assessments based on z‐scores or caterpillar plots.

4 The exponential form of the weights comes from the logistic link of model (4).

## Appendix 1

Below, we describe a MCMC algorithm to impute *k* ordinal components by sampling from the posterior normal distribution (using a latent normal approach). Because each component may have a different missingness pattern, the appropriate conditional distribution will have to be derived for each component *k*, given all other components, *Z* ^*K* − 1^, with *k* = 1, …, *K*. While the Gibbs sampler can be used (Albert and Chib, 1993), the more general Metropolis–Hastings sampler typically results in faster convergence (Cowles, 1996; Goldstein *et al*., 2007) and is considered here. This is particularly advantageous for estimating the threshold parameters (***α***) and updating level 1 and level 2 covariance matrices (**Ω**_*e*_, **Ω**_*u*_). The algorithm proceeds as follows. At each iteration *r* = 1, 2, …

Step 1. The first step is concerned with generating each latent normal variable, *Z* ^*k*^, given the observed ordinal component, *h* ^*k*^. For each component *k*
(a) Sample category 0 from a standard normal distribution [−∞, *α*_1_ − *ẑ*], categories 1, 2 and 3 from [*α*_*m* − 1_ − *ẑ*, *α*_*m*_ − *ẑ*] when *m* = 2, …, *M* − 1, and category 4 from [*α*_*M* − 1_ − *ẑ*, + ∞], where $\hat{z}=\hat{\beta }X+\hat{\delta }Z^{K-1}+\hat{u}$.
(b) Update the threshold parameters *α*_1_, …, *α*_*M* − 1_. Let the component of the likelihood associated with a specific category be given by $L\left(\alpha_{1},\ldots ,\alpha_{M-1}\right)=\Pi_{i=1}^{N}\Pi_{m=1}^{M}\pi_{m}^{I_{i,m}}$, where *I*_*i*,*m*_ is 1 if *h*_*ij*_ = *m* − 1, 0 otherwise, and

$$\pi_{m}=\left\{\begin{array}{c} \int_{-\infty }^{\alpha_{1}-\hat{z}}\varphi \left(t\right)dt,\;\text{if}\;m=1\;\\ \int_{\alpha_{m-1}-\hat{z}}^{\alpha_{m}-\hat{z}}\varphi \left(t\right)dt,\;\text{if}\;m=2,\ldots ,M-1\;\\ \int_{\alpha_{M-1}-\hat{z}}^{\infty }\varphi \left(t\right)dt,\;\text{if}\;m=M \end{array}\right.\;\text{.}$$

For each *α*_1_, …, *α*_*M* − 1_, we draw a proposal $\hat{\alpha }\sim N\left(\alpha^{r-1},v^{2}\right)$ and accept the proposal with probability min $\left[1,\;\frac{L\left(\tilde{\alpha }\right)}{L\left(\alpha^{r-1}\right)}\right]$. If $\tilde{\alpha }$ is accepted, set $\alpha_{m}^{*}=\tilde{\alpha }$, otherwise *α* ^*r*^ = *α* ^*r* − 1^. In practice, the variance of the proposal distribution is typically set to *v* ^2^ = 5.8/*N* (Gelman *et al*., 2006).

Step 2 Draw the missing values conditional on the values drawn for ***α*** ^*r*^ and ***Z*** ^*r*^; for each missing observation *h*_*ij*_, impute *h*_*ij*_ = *m* − 1 by finding the value such that $\alpha_{m-1}^{*}<Z_{ij}^{r}\leq \alpha_{m}^{*}$.
Step 3 Draw **β** ^*r*^ from $\left[{\left[\sum_{ij}\Lambda^{T}\Omega_{e}^{-1}\Lambda \right]}^{-1}\sum_{ij}\Lambda^{T}\Omega_{e}^{-1}{\left(Z_{ij}-u_{j}\right)}^{T},\;{\left[\sum_{ij}\Lambda^{T}\Omega_{e}^{-1}\Lambda \right]}^{-1}\right]$, where **Λ** = **I**_*k* × *k*_ ⊗ ***X***_*ij*_.
Step 4 Sample $u_{j}^{r}$ from $MVN\left[{\left[\sum_{i}\Omega_{e}^{-1}+\Omega_{u}^{-1}\right]}^{-1}{\sum_{i}\Omega_{e}^{-1}\left(Z_{ij}-\beta X_{ij}\right)}^{T},\;{\left[\sum_{i}\Omega_{e}^{-1}+\Omega_{u}^{-1}\right]}^{-1}\right]$. Level 1 residuals can be easily obtained by subtraction, $e_{ij}^{r}=Z_{ij}-\Lambda \beta -u_{j}$.
Step 5 Update the elements of $\Omega_{e}^{r-1}$ and $\Omega_{u}^{r-1}$, in that order and conditional on (***α*** ^*r*^, ***Z*** ^*r*^, ***β*** ^*r*^, ***u*** ^*r*^), to obtain $\Omega_{e}^{r}$ and $\Omega_{u}^{r}$ (Browne, 2006).
