# Supplementary material for: Addressing Missing Data in Patient‐Reported Outcome Measures (PROMS): Implications for the Use of PROMS for Comparing Provider Performance
Source: Health Econ. 2015 Mar 5;25(5):515–28. doi: 10.1002/hec.3173 (PMC4973682; doi:10.1002/hec.3173)
Supplement: Supplementary file 1 — Supporting info item [file HEC-25-515-s001.zip › Appendix A2.docx]

**Addressing missing data in PROMs using EQ-5D**

The EQ-5D is a widely used generic measure of health-related quality of life that describes impairments in overall health through self-assessed limitations on mobility, self-care and usual activities as well as pain/discomfort, and anxiety/depression. Each of the five dimensions, can take the value 1, indicating no problems, 2 for some/moderate problems and 3 for extreme problems. A patient’s health profile can then be described as a series of numerical values, e.g. 11221 representing a patient that has some problems performing usual activities and experiences moderate pain or discomfort but reports no problems on any other health dimension. The EQ-5D health profile can be aggregated to an index score using a UK-specific set of weights. These weights are derived from the general public and reflect societal preferences. The resulting index scores range from -0.594 to 1, where one is defined as perfect health and zero is defined as equivalent to being dead. Values lower than zero indicate health states that are considered worse than being dead.

As with Oxford Hip Score, we addressed the missing data in the individual dimensions of the EQ-5D. The observed predictors of missing EQ-5D were similar to those of Oxford Hip Score, and hence, we considered the same imputation models as before (implemented separately for Q1 and Q2). After imputation, we combined the individual components into an overall EQ-5D score using the UK-specific weights. We considered the same approach (section 3.1 in the paper) for estimating provider-specific outcomes.

The results are presented below. Figure 1 shows the distribution of case-mix adjusted post-operative EQ-5D for the complete-case analysis and multiple imputation. Figure 2 illustrates provider performance status via the funnel plots for complete cases and multiple imputation, with volume and outcome effects separated out. Table 1 reports the number of providers under each performance category for the alternative approaches.

**Figure A2.1.** Kernel density of the adjusted post-operative EQ-5D for complete case analysis (CCA) versus multiple imputation (MI).

**Figure 3.** Funnel plots of provider-specific outcomes (EQ-5D) according to complete cases (N=278), and after multiple imputation volume and mean outcome effects (N=298).

**Table A2.1.** Provider performance status according to EQ-5D for complete-case analysis and multiple imputation (MI).

|  | **Complete cases** | | **MI (volume effect)** | | **MI (Volume + outcome effects)** | |
| --- | --- | --- | --- | --- | --- | --- |
|  | **N** | **%** | **N** | **%** | **N** | **%** |
| Negative *alarm* | 15 | 5.4 | 35 | 11.7 | 32 | 10.7 |
| Negative *alert* | 17 | 6.1 | 21 | 7.1 | 21 | 7.1 |
| *In control* | 220 | 79.1 | 194 | 65.1 | 190 | 63.8 |
| Positive *alert* | 18 | 6.4 | 30 | 10.1 | 34 | 11.4 |
| Positive *alarm* | 8 | 2.9 | 18 | 6.0 | 21 | 7.1 |
| Total | 278 | 100.0 | 298 | 100.0 | 298 | 100.0 |
